# Altered Intracellular ATP Production by Activated CD4+ T-Cells in Very Preterm Infants

**DOI:** 10.1155/2016/8374328

**Published:** 2016-12-13

**Authors:** Giulia Aquilano, Maria Grazia Capretti, Francesca Nanni, Luigi Corvaglia, Arianna Aceti, Liliana Gabrielli, Angela Chiereghin, Giacomo Faldella, Tiziana Lazzarotto

**Affiliations:** ^1^Department of Obstetrical, Gynecological, and Pediatric Sciences, Operative Unit of Neonatology, St. Orsola-Malpighi University Hospital, University of Bologna, Bologna, Italy; ^2^Department of Specialized, Experimental, and Diagnostic Medicine, Microbiology, St. Orsola-Malpighi University Hospital, University of Bologna, Bologna, Italy

## Abstract

*Background*. The neonatal immune system is not fully developed at birth; newborns have adequate lymphocytes counts but these cells lack function.* Objective*. To assess the activity of T-cells and the influence of the main perinatal factors in very preterm infants (birth weight < 1500 g).* Design*. Blood samples from 59 preterm infants (21/59 were dizygotic twins) were collected at birth and at 30 days of life to measure CD4+ T-cell activity using the ImmuKnow™ assay. Fifteen healthy adults were included as a control group.* Results*. CD4+ T-cell activity was lower in VLBW infants compared with adults (*p* < 0.001). Twins showed lower immune activity compared to singletons (*p* = 0.005). Infants born vaginally showed higher CD4+ T-cell activity compared to those born by C-section (*p* = 0.031); infants born after prolonged Premature Rupture of Membranes (pPROM) showed higher CD4+ T-cell activity at birth (*p* = 0.002) compared to infants born without pPROM. Low CD4+ T-cell activity at birth is associated with necrotizing enterocolitis (NEC) in the first week of life (*p* = 0.049).* Conclusions*. Preterm infants show a lack in CD4+ T-cell activity at birth. Perinatal factors such as intrauterine inflammation, mode of delivery, and zygosity can influence the adaptive immune activation capacity at birth and can contribute to exposing these infants to serious complications such as NEC.

## 1. Introduction

Although the immune system development begins early during fetal life, its maturation is not completed at birth as confirmed by the increased susceptibility of newborns and preterm infants to infectious diseases. The immune system of the fetus/newborn protects the infant against infection at the maternal-fetal interface but must also avoid the potentially harmful proinflammatory/Th1-cell response that can induce a dangerous reaction between mother and fetus. Thus, the suppression of proinflammatory response helps the infant in the transition from the intrauterine environment to the foreign antigen-rich environment of the outside world. Therefore this ineffectiveness that has been interpreted for a long time as a deficiency of the immune system could represent a biologically advanced response.

The faults of the neonatal immune system are mostly related to a functional deficit of their components. Newborns and especially preterm infants have higher leukocyte and lymphocyte counts compared to adults [1]; however these cells show poor function at birth as a consequence of the adaptive immune system not having had strong antigenic exposure* in utero.* This is confirmed by the decreased number of memory T- and B-cells and the increased number of naïve T- and B-cells in the neonatal bloodstream [2].

In order to measure the immune cell function during the third trimester of gestation, the immune status in a group of preterm infants was evaluated at birth and after 30 days of life using the ImmuKnow assay (Viracor-IBT Laboratories, Lee's Summit, MO). This immunological test measures the levels of intracellular adenosine triphosphate (iATP) after* in vitro* nonspecific stimulation with phytohemoagglutinin (PHA) as marker of CD4+ T-cell activity; therefore it is one of the methods for non-pathogen-specific immune monitoring. Exposing T-cells to a mitogenic stimulus such as PHA leads to their metabolic activation and polyclonal expansion, a process in which the ATP synthesis and release precede surface receptor expression, cytokine production, and other subsequent events. Therefore, the iATP levels offer a proxy for the degree of functionality of the cell-mediated immune response [3]. The ImmuKnow assay has been cleared by the US Food and Drug Administration in 2002 for measuring changes in cell-mediated immunity in solid organ transplant recipients undergoing immunosuppressive therapy [4] and now represents one of the few well-established strategies for functional immune monitoring in the above-mentioned patient population [3].

Despite that, there are limited reports on the use of ImmuKnow assay in a pediatric population. Hooper et al. tested ImmuKnow assay in healthy children aged 1–17 years. The authors found that children aged 12 years and older have immune function levels equal to adults, while children aged less than 12 years present lower immune function levels compared to adults and older children [5].

However, to the best of our knowledge, ImmuKnow assay has never been tested in newborns and preterm infants.

The aim of this study was to estimate the peripheral blood CD4+ T-cell activation rate in response to* in vitro* stimulation with PHA in order to assess the basal condition of the adaptive immune system at birth, its development in the first month of life, and the influence of the main perinatal factors on the immune response in a population of very preterm infants.

## 2. Methods

### 2.1. Population

A prospective longitudinal study was carried out between November 2013 and July 2015 at the Neonatal Intensive Care Unit (NICU) of St. Orsola-Malpighi University Hospital in Bologna, Italy.

All the infants with gestational age (GA) ≤ 30 weeks and birth weight (BW) < 1500 g admitted at birth to the NICU were considered eligible to the study. Infants with congenital malformations or congenital infections or born to a mother with pregnancy complications (immunosuppressive disorders, diabetes mellitus, or infections during or preexisting the pregnancy) were excluded. Before enrollment in the study, written informed consent was obtained from each of the infants parents.

Fifteen healthy adults were also included as a control group.

### 2.2. Study Design

Peripheral blood samples were collected in the first day of life and at 30 days of life from each patient to evaluate the pattern of lymphocytes subpopulations and the levels of iATP in activated CD4+ T-cells* in vitro.* Anamnestic and clinical data were prospectively collected during the hospital stay.

Sepsis was defined as presence of clinical signs of infection (worsening of respiratory dynamics, apnea and increased oxygen requirement, cardiovascular instability with tachycardia or bradycardia, poor perfusion, hypotonia, and shock), elevation of infections markers (white cell count, CRP, and procalcitonin), and ≥1 positive blood cultures.

Necrotizing enterocolitis (NEC) was defined according to Bells modified criteria [6].

The study was approved by the St. Orsola-Malpighi University Hospital Research Ethics Committee (CIMPre study, 114/2012/U/Oss).

### 2.3. Assessment of CD4+ T-Cell Activity and Lymphocyte Subsets

CD4+ T-cell immune response was measured using the ImmuKnow assay (Viracor-IBT Laboratories, Lee's Summit, MO). The test was performed at the Operative Unit of Clinical Microbiology—Laboratory of Virology—of St. Orsola-Malpighi University Hospital in Bologna, Italy, according to the package insert.

Peripheral blood was collected in sodium heparin tubes and processed on the same day. Briefly, 250 *μ*L of whole blood was diluted with sample diluent, added to wells of a 96-well microtiter plate (12 × 8-well separable strips; one 8-well strip/each specimen), and incubated for 15–18 h with PHA in 37°C in 5% CO_2_. Magnetic particles coated with anti-human CD4 antibodies were introduced to the wells, and using a strong magnet, CD4+ T-cells were positively selected and separated. Then, the cells were lysed to release intracellular ATP. The released iATP was measured using luciferin/luciferase reagent and a luminometer.

The patient's CD4+ T-cell immune response was expressed as the amount of iATP (ng/mL).

In order to check each assay run, a control specimen collected from a healthy adult individual (quality control sample) was processed with patient specimen. Quality control samples were not included in the adult control group.

The lymphocyte subsets were evaluated using the BD FACSCalibur Flow Cytometr (BD FACSCaliburTM system, Becton & Dickson, Mountain View, CA, USA) according to the manufacturer's instructions.

### 2.4. Statistical Analysis

Statistical analysis was performed by IBM SPSS (Statistical Package for Social Sciences, version 20). Data distribution was checked for normality with the Shapiro-Wilk test. As the data is not normally distributed, nonparametric tests were used. Univariate analysis was performed in order to evaluate which clinical variables were related to iATP values at birth and at one month of life: Mann–Whitney and Kruskall-Wallis tests were used for categorical variables and Spearman correlation test for continuous variables. Regression multivariate analysis was performed using as independent variables all those variables that proved to be significant in the univariate analysis. Statistical significance was defined as a *p* value < 0.05.

## 3. Results

Seventy-three eligible infants were admitted to NICU during the study period. Fourteen infants (19,1%) were excluded because they either fulfilled the exclusion criteria (4 cases of congenital heart disease and 4 cases of polymalformations) or peripheral blood samples could not be collected within the first day of life (4 infants died and 2 were transferred from our unit in the first days of life). The study was carried out in a population of 59 preterm infants with GA ≤ 30 weeks and BW < 1500 g; 21/59 were dizygotic twins (the study population does not include monozygotic twins).

Samples were obtained at 30 days of life from 39/59 (66.1%) of the recruited newborns; the remaining 20/59 (33.8%) encountered death (4 infants) or were transferred to other hospitals before 30 days of life.

### 3.1. Lymphocyte Subsets

The pattern of lymphocytes subpopulations at birth and at 30 days of life is reported in Table 1. We found no significant difference between the absolute cell count at birth and at the end of the first month of life. While the absolute number of CD4+ T-cells did not correlate to the median value of iATP at birth (*p* = 0.831) a significant positive correlation was observed at 30 days of life (*p* = 0.011).

### 3.2. CD4+ Activity in Preterm Newborns at Birth and 30 Days of Age Compared to Adult Controls

**Figure 1:**
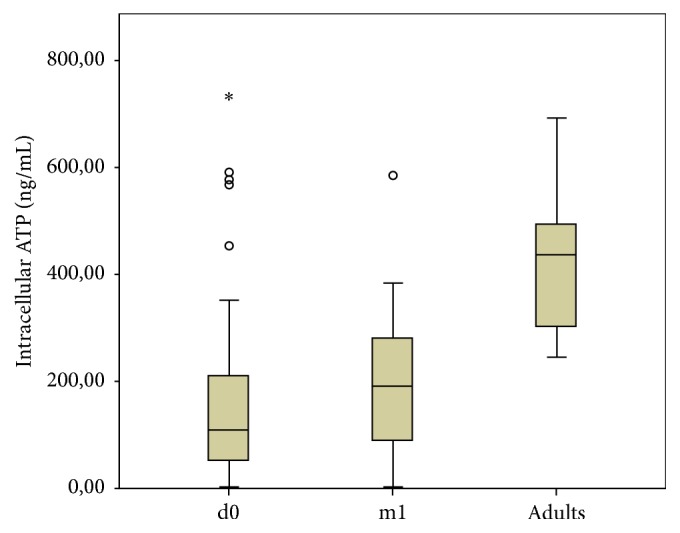
CD4+ T-cell activity (median iATP values) in preterm infants and in adult controls (d0: evaluation at birth; m1: evaluation at 30 days of life). Outliers are marked as ∘ (“out values”: 1,5–3 × interquartile range, IQR) and *∗* (“extreme values”: > 3 × IQR).

While there were no significant differences in CD4+ T-cells ATP levels at birth versus 30 days of life (median: 100 ng/mL [range: 6–733 ng/mL] versus 168 ng/mL [range: 3–383 ng/mL]; *p* = .142), both these values were significantly lower compared to adults controls (*p* < 0.001) as shown in Figure 1.

### 3.3. Perinatal Factors and CD4+ Activity

Univariate analysis showed no significant correlation between median ImmuKnow iATP levels at birth and GA, BW, gender, intrauterine growth retardation (SGA, small for gestational age), and use of prenatal steroids (Table 2).

**Figure 2:**
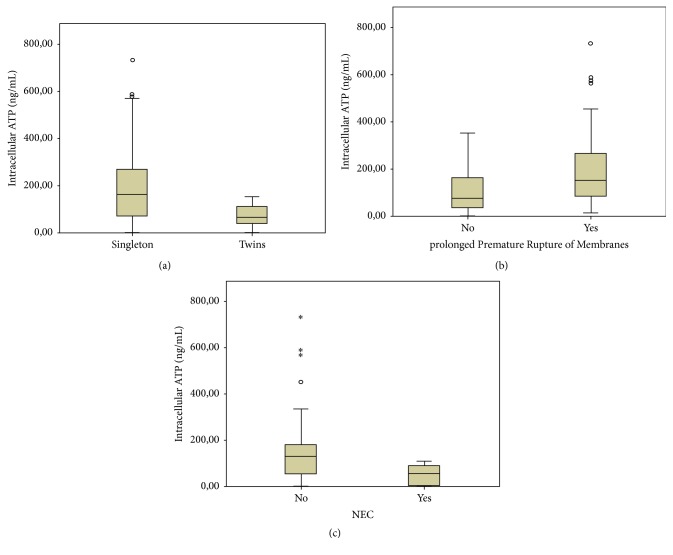
Levels of ATP at birth: (a) in twins and singleton; (b) pPROM versus other conditions of delivery; (c) patients with and without NEC. Outliers are marked as ∘ (“out values”: 1,5–3 × interquartile range, IQR) and *∗* (“extreme values”: > 3 × IQR).

The twenty-one twins showed significantly lower median ImmuKnow iATP levels at birth compared to the remaining 38 singleton infants (*p* = 0.005); this difference was no longer significant at 30 days of life (Table 2 and Figure 2(a)).

Infants born to vaginal delivery had higher median ATP levels produced by activated CD4+ T-cells at birth compared to those born to C-section (*p* = 0.031); no difference was found at 30 days of life (Table 2).

Fifteen out of 59 preterm infants were born to mothers with pPROM (prolonged Premature Rupture of Membranes > 18 hrs); these infants showed a higher activity of CD4+ T-cells at birth compared to the 44 remaining infants (*p* = 0.002); this difference disappeared at 30 days of life (Table 2 and Figure 2(b)).

A multivariate analysis was performed including those variables (pPROM and twin pregnancy) which proved to be significant in the univariate analysis: both pPROM (Standardized Beta = 0.344, *t* = 2.875,  and *p* = 0.006) and twin pregnancies (Standardized Beta = −0.359, *t* = −3.008,  and *p* = 0.004) were found to be independently associated with ImmuKnow iATP levels at birth (higher and lower values, resp.).

### 3.4. Morbidity during the First 30 Days of Hospital Stay and CD4+ Activity

No correlation was found between CD4+ T-cell activity at birth or at 30 days of life and mechanical ventilation, patency of ductus arteriosus, early onset sepsis, late onset sepsis, postnatal steroids, antibiotics use, type of enteral nutrition, and death.

Five out of 59 (8.5%) preterm infants developed NEC ≥ stage 3 during the first week of life; these infants had significantly lower median iATP values at birth compared to the 55 infants without NEC (*p* = 0.049) (Figure 2(c)). We also performed a subgroup analysis dividing the infants into three subgroups according to gestational age (23-24, 25–27, and 28–30 weeks). We found that three out 5 infants who developed NEC were in the 23-24 weeks subgroup and the remaining two were in the 25–27 weeks one. In the 23-24 weeks group, there was still a significant difference in terms of immune cell function between infants who did and did not develop NEC (*p* = 0.036). On the contrary, the difference of median iATP levels between infants who did and did not develop NEC in the 25–27 weeks group was not significant, but we can not exclude the possibility that this is a consequence of the little data we had available.

No difference in CD4+ T-cell activity was found at 30 days of life in relation to NEC condition.

### 3.5. Short-Term Outcomes and CD4+ Activity

We found no influence of CD4+ T-cell activity on short-term outcomes (IVH: intraventricular hemorrhage; PVL: periventricular leukomalacia; ROP: retinopathy of prematurity; BPD: Bronchopulmonary Dysplasia; death), at birth or at 30 days of life.

## 4. Discussion

The neonatal immune system is not fully developed at birth and newborns are therefore exposed to the risk of infection by a large number of viruses, bacteria, protozoa, and fungi. This weakness can be partly attributed to the lack of preexisting immunological memory and competent adaptive immunity. Newborn infants have deficiencies in T-cell activation and cytokine production, B-cell immunoglobulin production, and interactions between T- and B-cells, when compared to adults [7]. T-lymphocytes, especially CD4+ T-cells, play a crucial role in the regulation of the immune system. They are often targeted as a marker of the global immune function because they are involved in the modulation of the adaptive immunity, both humoral and cell-mediated, and they also play an important role in the control of innate immune response.

From 19 weeks of gestation, T-cell subpopulations gradually increase in number and continue to rise after birth to peak at about 6–9 months of life. The numbers subsequently decline, to reach adult levels at 6-7 years of age [8]. In term neonates, CD4+ T-cells constitute a higher proportion of T-cells than adults. CD8+ T-cells on the other hand are fewer both in terms of absolute number and as a percentage of total T-cells. Preterm infants have a significantly higher number of CD4+ T-cells while the number of CD8+ T-cells does not seem to change with gestational age [9]. Reference values for T-lymphocyte count are established for all ages but they do not reflect cell function.

In this study, we used the ImmuKnow assay in order to investigate the function of CD4+ T-cells at birth in a population of preterm infants. This test is one of the methods for non-pathogen-specific immune monitoring and measures the levels of ATP produced by CD4+ T-cells in response to* in vitro* stimulation with a nonspecific mitogen such as PHA [4]. ATP production is the final response of CD4+ cells that is common to different nonspecific factors. ATP is a key metabolic marker; it is produced within minutes to hours of initial stimulation and is necessary for cellular function regardless of eventual effector function and therefore it is a highly suitable marker for T-lymphocyte activation and a clinical evaluation of global T-lymphocyte function [10]. At this moment there are no certainties regarding if the low ATP production by premature CD4 cells is due to inherent immaturity of ATP production, signaling, immature proliferation mechanism, or something else. However, some authors [11–15] suggest that the inability of neonatal CD4+ T-cells to go towards an efficient activation may be related/caused by an impaired process of signaling. A limit of the ImmuKnow assay is the lack of specificity, because PHA cause a nonspecific mitogenic stimulation that can not define any specific aspect of the immune response, like cytokine expression that marks Th1 or Th2 activation; but its major advantage is that it is clinically available unlike most other immunological assays. ImmuKnow assay has previously only been tested in adults and children and this is the first study that uses this assay to assess T-cell-mediated immunity in preterm infants. This test seemed quite suitable for preterm infants since it requires a very small amount of peripheral blood to be performed (250 *μ*L).

Our findings show similar leukocyte and CD4+ T-cells counts to previously reported studies in preterm population [2, 16].

We documented a lack of association between the number of CD4+ T-cells at birth and their function (measured by intracellular ATP production) and a positive correlation between number and activity of CD4+ T-cells at 30 days of life. Whether there is or there is no correlation between the number of lymphocytes and their function is still a matter of debate in literature.

Kowalski et al. [10] demonstrated that this correlation is weak (*r* = 0.24), emphasizing the usefulness of ImmuKnow assay to provide a quantification of CD4+ T-cells activity that is independent of cells absolute number.

However, other studies performed afterwards questioned this observation since a positive association between white blood cell count and ImmuKnow levels was observed [17, 18]. Dharnidharka and Hesemann suggest that this may represent an underappreciated limitation of the ImmuKnow test since the levels of ATP are expressed as a concentration (ng/mL) without accounting for the number of white blood cells present in the volume of sample tested [19].

Because each study is heterogeneous from the other, differing in characteristics such as population, type of organ transplant, immunosuppression protocols, and timing of ImmuKnow assay it is difficult to come to conclusions.

We hypothesized that the correlation in our study is initially absent as a consequence of the immunological immaturity at birth (lymphocytes may be present in the blood without being functional) and becomes significant over time with the functional maturation of lymphocytes. Other neonatal confounding factors may also play a role and cannot be excluded and larger studies on immunocompetent subjects may validate our hypothesis.

In our study the adult control population includes healthy HIV-seronegative immunocompetent people, whose lymphocytes subpopulations related data are not available.

We found that preterm infants have a reduced CD4+ T-cell activation capacity compared to adults: the iATP values at birth are low, and despite a trend towards higher values over time, they remain significantly lower at 30 days of life. It is known that newborns, especially preterm infants, have deficiencies in both innate and adaptive immunity and many studies have demonstrated lower concentrations of cytokines such as TNF-*α*, IFN-*α*, IL-4, IL-5, IL-10, IL-15, and IFN-*γ* in preterm infants' blood compared to adults [20–23]. However cytokines production is an indirect estimate of cellular function. The present study sets the functional impairment of CD4+ T-cells at the initial steps of T-lymphocyte activation, when ATP is produced. Since ATP is a basic energy source within cells, its production is an essential requirement for all lymphocytes function following activation [10].

In this study twin infants showed median ImmuKnow iATP levels at birth much lower compared to singletons. This is an interesting find and literature lacks information on the immunological peculiarities of multiple pregnancies. All the twins included in the study were dizygotic and thus immunologically different. We hypothesize that, in addition to a generalized status of immunological deficiency or insufficiency, the copresence of twins in utero may induce a deeper immune tolerance that involves both fetuses and the mother in order to avoid the potentially harmful immune reaction between the three. Our hypothesis is in accordance with previous studies that documented higher levels of Th2-cytokines in the blood of mothers carrying twins compared with singleton pregnancies [24] underlining the more profound Th1-Th2 shift that occurs in twin pregnancies. Moreover, a recent study demonstrated that both dizygotic twins and their mothers are more prone to infection than monozygotic twins, singletons, and their mothers [25].

We found that pPROM significantly increases CD4+ T-cells activation at birth. This data is consistent with previous findings that reported that lymphocytes are activated during infections in utero, indicating that fetal adaptive immune response is at least partly responsive [26]. It has been shown that bacteria and proinflammatory mediators in amniotic fluid can elicit a fetal inflammatory response, documented by an increase in fetal plasma cytokines and C reactive protein [27–29]. Fetuses and neonates exposed to intrauterine inflammation have increased Th1 cells response and increased levels of IFN-*γ*, indicating a potential shift from Th2 to Th1 of the fetus [7, 30].

We have also demonstrated that CD4+ T-cell activity at birth is increased in infants born after a vaginal delivery. Increasing evidence suggests that parturition itself is an inflammatory event [31].

Our findings support the current knowledge that intrauterine inflammation/infection can lead to immune maturation in the fetus. But these differences disappear at 30 days of life most likely because all the categories (pPROM versus non-pPROM and vaginal delivery versus cesarean section) are exposed to the same extrauterine environment.

In our study population, the levels of iATP at birth did not correlate with the risk of sepsis. This is not the first study that fails to detect an association between low ImmuKnow iATP values and infection. Regarding this topic, previous studies performed in immunocompromised adults showed conflicting results [32]. In fact in the transplant setting, some authors reported that the ImmuKnow assay was not able to identify individuals at risk of infection, while others found that the assay was a useful tool for assessing this risk [33, 34].

Significant positive correlations were observed between low CD4+ T-cell activation capacity at birth and NEC development in the first week of life. Although the etiopathogenesis of NEC is still a matter of debate, some authors support the involvement of innate immune system [35]. We hypothesize that an impairment of the adaptive immune system may also play a role in the altered immune reaction that leads to NEC development. CD4+ T-cells impaired function may hinder infection control in the intestinal lumen. Other mechanisms may also be involved and a more detailed characterization of CD4+ T-cells is needed to clarify their role in the NEC pathogenesis.

This study has some limitations. The study sample is small and lacks control groups both at term and with gestational age > 30 weeks.

Another limitation is the absence of an immunophenotypic classification of CD4+ T-cells. The evaluation of Treg cells is crucial to understanding the immunological characteristics of the preterm infants. These CD4+ T-cells are provided with immunosuppressive functions and represent a high proportion of lymphocytes at birth with a significant inverse correlation with GA [9]. The role of Treg cells in the modulation of the immune response is an expanding field of research and this data can add precious information to our findings.

In conclusion, preterm infants show a lack in CD4+ T-cells activation and fail to show a functional maturation of lymphocytes over the first month of life. An impaired ability to respond to stimulation can contribute to expose these infants to serious complications such as NEC. However, the adaptive immune response can at least partially be elicited during the fetal life by events occurring before delivery such as pPROM or vaginal delivery. Further studies in larger populations are needed to clarify these results and to better understand the cellular mechanism that regulates neonatal adaptive immune response to pathogens.

## Figures and Tables

**Table 1:** Lymphocytes subpopulations at birth and at 30 days of age of infants enrolled in the study (WBC: white blood cells, N: neutrophils, L: lymphocytes, and NK: natural killer lymphocytes).

	Birth: 59 infants Median (range)	30 days: 39 infants Median (range)
WBC (n)	7350 (1170–119200)	10700 (4030–34650)
N (n)	1814 (283–97744)	4267 (908–23562)
N (%)	24.8 (4.3–88)	42 (8.9–75)
L (n)	3775 (903–10775)	4141 (557–10098)
L (%)	60 (6–86.8)	40 (4.4–74)
Pan T (CD3+)	2875 (668–8404.5)	2865 (701–5481)
Pan T (%)	73.9 (47–88)	64.5 (36–84)
CD4+/mL	1954 (388–6680.5)	1982 (438–4725)
CD4+ (% L tot)	54 (32–73)	45.5 (20–69)
CD8+ mL	673 (172–2312)	729.5 (198–2650.5)
CD8+ (% L tot)	18 (8–32)	16 (8–45)
CD4+/CD8+	2.92 (1.37–7.38)	2.67 (0.77–6.25)
NK/mL	242.25 (35.6–1536)	442.75 (15–1371)
NK (% L tot)	7 (1–24)	9.5 (1–42)
Pan B/mL	501 (60.56–3770)	829.35 (210–3635.3)
Pan B (%)	15 (2–30.6)	22.9 (9–41)

**Table 2:** Characteristics at birth of infants enrolled in the study: median (range); GA: gestational age; SGA: small for gestational age; AGA: appropriate for gestational age; ^*∗∗∗*^pPROM: prolonged Premature Rupture of Membranes: a case of premature rupture of membranes in which more than 18 hours has passed between the rupture and the onset of labor/delivery.

	Birth (59 infants)	*p* value	30 days of life (39 infants)	*p* value
Gestational age, weeks, mean ± DS	27.7 ± 2.4		27.5 ± 2.2	
Birth weight, mean ± DS (g)	992 ± 297		981 ± 281	
Gender male, infants number (%)	33 (55.9)		23 (59.0)	
Male sex, mean ± DS (iATP ng/mL)	182 ± 157	*p* = 0.091	170 ± 123	*p* = 0.326
Female sex, mean ± DS (iATP ng/mL)	110 ± 140.6		187 ± 105	
Singleton, infants number (%)	38 (64.4)		25 (64.1)	
Singleton: iATP ng/mL^∧^	163 (6–733)	*p* = 0.005	178 (3–383)	*p* = 0.731
Twins: iATP ng/mL^∧^	84 (26–153)		168 (10–365)	
Small for GA, infants number (%)	10 (16.9)		6 (15.4)	
SGA, mean ± DS (iATP ng/mL)	106 ± 121.1	*p* = 0.255	175 ± 96.0	*p* = 0.770
AGA, mean ± DS (iATP ng/mL)	164 ± 158		177 ± 120	
Vaginal delivery, infants number (%)	28 (47.5)		19 (48.7)	
Vaginal delivery: iATP ng/mL^∧^	123 (15–733)	*p* = 0.031	123.5 (3–383)	*p* = 0.795
Cesarean delivery: iATP ng/mL^∧^	83 (6–352)		215 (3–292)	
pPROM^*∗∗∗*^, infants number (%)	15 (25.4)		9 (23.0)	
pPROM^*∗∗∗*^: iATP ng/mL^∧^	197 (52–336)	*p* = 0.002	185.5 (65–383)	*p* = 0.343
All others conditions: iATP ng/mL^∧^	87 (6–733)		168.5 (3–365)	
Prenatal steroids, infants number (%)	48 (81.3)		33 (84.6)	
Prenatal steroids, mean ± DS (iATP ng/mL)	169 ± 162	*p* = 0.140	189 ± 116	*p* = 0.159
No prenatal steroids, mean ± DS (iATP ng/mL)	79 ± 52		117 ± 95	

^∧^ means that the values are expressed as median and range (brackets) instead of mean ± standard deviation.

## References

[1] Quinello C., Silveira-Lessa A. L., Ceccon M. E. J. R., Cianciarullo M. A., Carneiro-Sampaio M., Palmeira P. (2014). Phenotypic differences in leucocyte populations among healthy preterm and full-term newborns. *Scandinavian Journal of Immunology*.

[2] Walker J. C., Smolders M. A. J. C., Gemen E. F. A., Antonius T. A. J., Leuvenink J., De Vries E. (2011). Development of lymphocyte subpopulations in preterm infants. *Scandinavian Journal of Immunology*.

[3] Fernández-Ruiz M., Kumar D., Humar A. (2014). Clinical immune-monitoring strategies for predicting infection risk in solid organ transplantation. *Clinical & amp; Translational Immunology*.

[4] Kowalski R., Post D., Schneider M. C. (2003). Immune cell function testing: an adjunct to therapeutic drug monitoring in transplant patient management. *Clinical Transplantation*.

[5] Hooper E., Hawkins D. M., Kowalski R. J. (2005). Establishing pediatric immune response zones using the Cylex® ImmuKnow™ assay. *Clinical Transplantation*.

[6] Kliegman R. M., Walsh M. C. (1987). Neonatal necrotizing enterocolitis: pathogenesis, classification, and spectrum of illness. *Current Problems in Pediatrics*.

[7] Melville J. M., Moss T. J. M. (2013). The immune consequences of preterm birth. *Frontiers in Neuroscience*.

[8] Erkeller-Yuksel F. M., Deneys V., Yuksel B. (1992). Age-related changes in human blood lymphocyte subpopulations. *The Journal of Pediatrics*.

[9] Sériès I. M., Pichette J., Carrier C. (1991). Quantitative analysis of T and B cell subsets in healthy and sick premature infants. *Early Human Development*.

[10] Kowalski R. J., Zeevi A., Mannon R. B., Britz J. A., Carruth L. M. (2007). Immunodiagnostics: evaluation of functional T-cell immunocompetence in whole blood independent of circulating cell numbers. *Journal of Immunotoxicology*.

[11] Miscia S., Di Baldassarre A., Sabatino G. (1999). Inefficient phospholipase C activation and reduced Lck expression characterize the signaling defect of umbilical cord T lymphocytes. *The Journal of Immunology*.

[12] Hii C. S. T., Costabile M., Mayne G. C., Der C. J., Murray A. W., Ferrante A. (2003). Selective deficiency in protein kinase C isoenzyme expression and inadequacy in mitogen-activated protein kinase activation in cord blood T cells. *Biochemical Journal*.

[13] Hassan J., Reen D. J. (1997). Cord blood CD4^+^ CD45RA^+^ T cells achieve a lower magnitude of activation when compared with their adult counterparts. *Immunology*.

[14] Pearce E., Pearce E. (2013). Metabolic pathways in immune cell activation and quiescence. *Immunity*.

[15] Pollizzi K. N., Powell J. D. (2014). Integrating canonical and metabolic signalling programmes in the regulation of T cell responses. *Nature Reviews Immunology*.

[16] Correa-Rocha R., Pérez A., Lorente R. (2012). Preterm neonates show marked leukopenia and lymphopenia that are associated with increased regulatory T-cell values and diminished IL-7. *Pediatric Research*.

[17] Sageshima J., Ciancio G., Chen L. (2014). Lack of clinical association and effect of peripheral WBC counts on immune cell function test in kidney transplant recipients with T-cell depleting induction and steroid-sparing maintenance therapy. *Transplant Immunology*.

[18] Ryan C. M., Chaudhuri A., Concepcion W., Grimm P. C. (2014). Immune cell function assay does not identify biopsy-proven pediatric renal allograft rejection or infection. *Pediatric Transplantation*.

[19] Dharnidharka V. R., Hesemann L. E. (2014). The ImmuKnow assay—does it really put us in the know about the immune system?. *Pediatric Transplantation*.

[20] Seghaye M.-C., Heyl W., Grabitz R. G. (1998). The production of pro- and anti-inflammatory cytokines in neonates assessed by stimulated whole cord blood culture and by plasma levels at birth. *Biology of the Neonate*.

[21] Chheda S., Palkowetz K. H., Garofalo R., Rassin D. K., Goldman A. S. (1996). Decreased interleukin-10 production by neonatal monocytes and T cells: relationship to decreased production and expression of tumor necrosis factor-*α* and its receptors. *Pediatric Research*.

[22] Qian J. X., Lee S. M., Suen Y., Knoppel E., van de Ven C., Cairo M. S. (1997). Decreased interleukin-15 from activated cord versus adult peripheral blood mononuclear cells and the effect of interleukin-15 in upregulating antitumor immune activity and cytokine production in cord blood. *Blood*.

[23] Lilic D., Cant A. J., Abinun M., Calvert J. E., Spickett G. P. (1997). Cytokine production differs in children and adults. *Pediatric Research*.

[24] Suzuki S., Okudaira S. (2004). Maternal peripheral T helper 1-type and T helper 2-type immunity in women during the first trimester of twin pregnancy. *Archives of gynecology and obstetrics*.

[25] Spiegler J., Härtel C., Schulz L. (2012). Causes of delivery and outcomes of very preterm twins stratified to zygosity. *Twin Research and Human Genetics*.

[26] Duggan P. J., Maalouf E. F., Watts T. L. (2001). Intrauterine T-cell activation and increased proinflammatory cytokine concentrations in preterm infants with cerebral lesions. *The Lancet*.

[27] Berry S. M., Romero R., Gomez R. (1995). Premature parturition is characterized by in utero activation of the fetal immune system. *American Journal of Obstetrics & Gynecology*.

[28] Goldenberg R. L., Hauth J. C., Andrews W. W. (2000). Intrauterine infection and preterm delivery. *The New England Journal of Medicine*.

[29] Gotsch F., Romero R., Kusanovic J. P. (2007). The fetal inflammatory response syndrome. *Clinical Obstetrics and Gynecology*.

[30] Sykes L., MacIntyre D. A., Yap X. J., Teoh T. G., Bennett P. R. (2012). The Th1:Th2 dichotomy of pregnancy and preterm labour. *Mediators of Inflammation*.

[31] Norman J. E., Bollapragada S., Yuan M., Nelson S. M. (2007). Inflammatory pathways in the mechanism of parturition. *BMC Pregnancy and Childbirth*.

[32] Rodrigo E., López-Hoyos M., Corral M. (2012). ImmuKnow as a diagnostic tool for predicting infection and acute rejection in adult liver transplant recipients: a systematic review and meta-analysis. *Liver Transplantation*.

[33] Ling X., Xiong J., Liang W. (2012). Can immune cell function assay identify patients at risk of infection or rejection? A meta-analysis. *Transplantation*.

[34] Ravaioli M., Neri F., Lazzarotto T. (2015). Immunosuppression modifications based on an immune response assay: results of a randomized, controlled trial. *Transplantation*.

[35] Tanner S. M., Berryhill T. F., Ellenburg J. L. (2015). Pathogenesis of necrotizing enterocolitis: modeling the innate immune response. *The American Journal of Pathology*.

